# Lin28 Mediates Radiation Resistance of Breast Cancer Cells via Regulation of Caspase, H2A.X and Let-7 Signaling

**DOI:** 10.1371/journal.pone.0067373

**Published:** 2013-06-20

**Authors:** Linbo Wang, Chao Yuan, Kezhen Lv, Shuduo Xie, Peifen Fu, Xiaojiao Liu, Yongxia Chen, Chuan Qin, Wuguo Deng, Wenxian Hu

**Affiliations:** 1 Key Laboratory of Biotherapy of Zhejiang Province, Department of Surgical Oncology, Sir Run Run Shaw Hospital, College of Medicine, Zhejiang University, Hangzhou, China; 2 Department of Breast Center, The First Affiliated Hospital, College of Medicine, Zhejiang University, Hangzhou, China; 3 Institute of Cancer Stem Cell, Dalian Medical University Cancer Center, Dalian, China; 4 State Key Laboratory of Oncology in South China, Sun Yat-Sen University Cancer Center, Guangzhou, China; University of South Alabama, United States of America

## Abstract

Resistance to radiation therapy is a major obstacle for the effective treatment of cancers. Lin28 has been shown to contribute to breast tumorigenesis; however, the relationship between Lin28 and radioresistance remains unknown. In this study, we investigated the association of Lin28 with radiation resistance and identified the underlying mechanisms of action of Lin28 in human breast cancer cell lines. The results showed that the expression level of Lin28 was closely associated with resistance to radiation treatment. The T47D cancer cell line, which highly expresses Lin28, is more resistant to radiation than MCF7, Bcap-37 or SK-BR-3 cancer cell lines, which have low-level Lin28 expression. Transfection with Lin28 siRNA significantly led to an increase of sensitivity to radiation. By contrast, stable expression of Lin28 in breast cancer cells effectively attenuated the sensitivity to radiation treatment. Stable expression of Lin28 also significantly inhibited radiation-induced apoptosis. Moreover, further studies have shown that caspases, H2A.X and Let-7 miRNA were the molecular targets of Lin28. Stable expression of Lin28 and treatment with radiation induced H2AX expression, while inhibited p21 and γ-H2A.X. Overexpression of Let-7 enhanced the sensitivities to radiation in breast cancer cells. Taken together, these results indicate that Lin28 might be one mechanism underlying radiation resistance, and Lin28 could be a potential target for overcoming radiation resistance in breast cancer.

## Introduction

It is estimated that more than 207,000 women in the United States will be newly diagnosed with breast cancer [1]. Although many anticancer therapies can alter tumor growth, in most cases the effect is not long lasting. Radiation therapy is widely used in breast cancer treatment, and its benefits have been studied extensively during the last decades. Clinical studies in women with breast cancer who undergo breast-conserving surgery have demonstrated that whole-breast irradiation could reduce the risk of local recurrence and improve survival outcomes [2]; [3]. However, resistance to radiotherapy is a major obstacle for the effective treatment of breast cancers. For some malignant tumors that are not sensitive to ionizing radiation, radiotherapy may not kill the tumor cells effectively. Thus, there is an urgent need to explore radiation resistance mechanisms to improve the sensitivity of radiotherapy.

Lin28 is a marker of cancer stem cells [4]. It is highly expressed in some tumors, such as hepatocellular carcinoma [5]. Overexpression of Lin28 has been shown to promote cancer cell proliferation [6]; [7]. There is a relationship between the expression of Lin28 and the radioresistance of lung cancer cells and pancreatic cancer cells [8]. However, no information is available to show a relationship between the dysregulation of Lin28 and the radioresistance of breast cancer cells. Moreover, the mechanism of action of Lin28 and its molecular targets are not completely understood.

Herein, we investigated the expression of Lin28 in various breast cancer cell lines and tumor tissues. We postulate that Lin28 expression is implicated in radioresistance. To investigate the underlying mechanisms, we analyzed the role of Lin28 in the regulation of HER2, H2AX, P21 and Let-7 miRNA. The present study suggests that Lin28 may be a potential target to overcome radioresistance and provides a scientific basis for further investigation of its mechanisms of radioresistance.

## Results

### Lin28 Expression is Associated with Radiation Resistance

**Figure 1:**
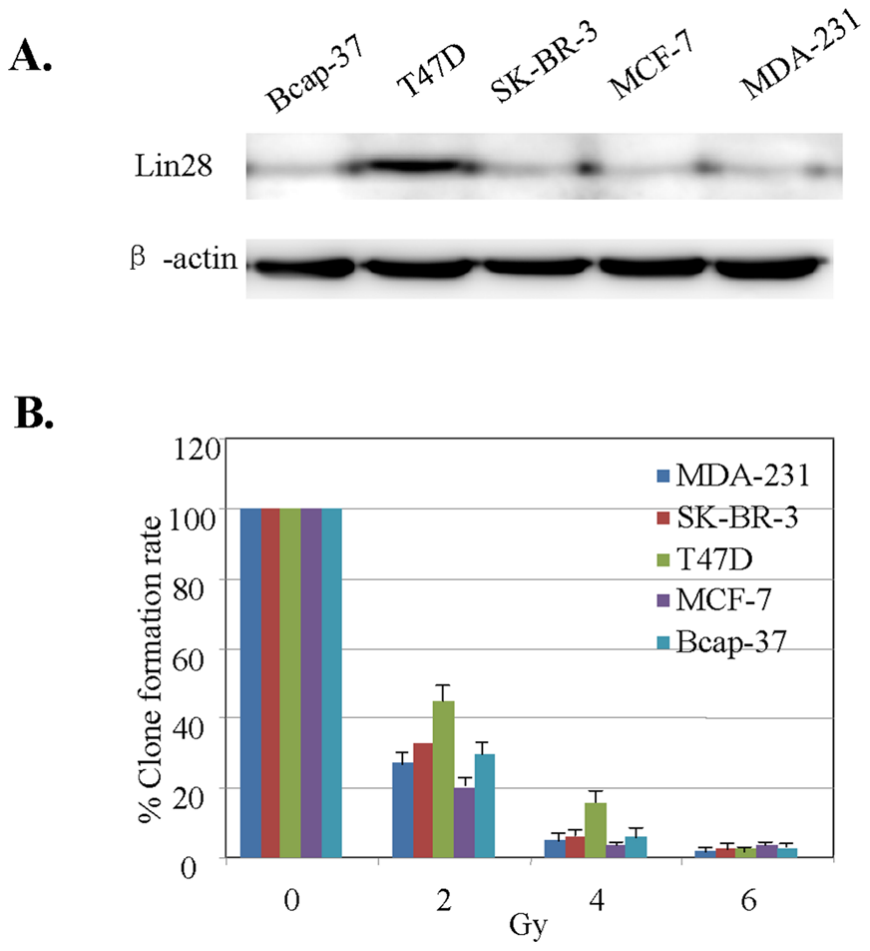
Lin28 expression is associated with sensitivity to radiation in breast cancer cells. (**A**). Lin28 expression was determined in the indicated breast cancer cell lines by Western blotting. β-actin expression was used as a loading control. (**B**). The cells were treated with different doses of radiation, and cell viability was determined by a clonogenic formation assay.

To determine whether Lin28 expression is associated with radioresistance in breast cancer, we measured the expression of Lin28 in five breast cancer cell lines and determined their sensitivities to the radiotherapy, which is used in breast cancer treatment. Our results showed that Lin28 was highly expressed in T47D cancer cells, whereas its expression was relatively lower in MCF7, Bcap-37 and SK-BR-3 MDA-231 cancer cells (Fig. 1A). The clonogenic formation assay showed that the survival rates of T47D cancer cells were 45% and 15% at doses of 2 Gy and 4 Gy of radiation treatment, respectively, whereas the rates of other cancer cells were much lower (Fig. 1B). These results indicate that high Lin28 expression increases the survival of T47D cells exposed to radiation.

**Figure 2:**
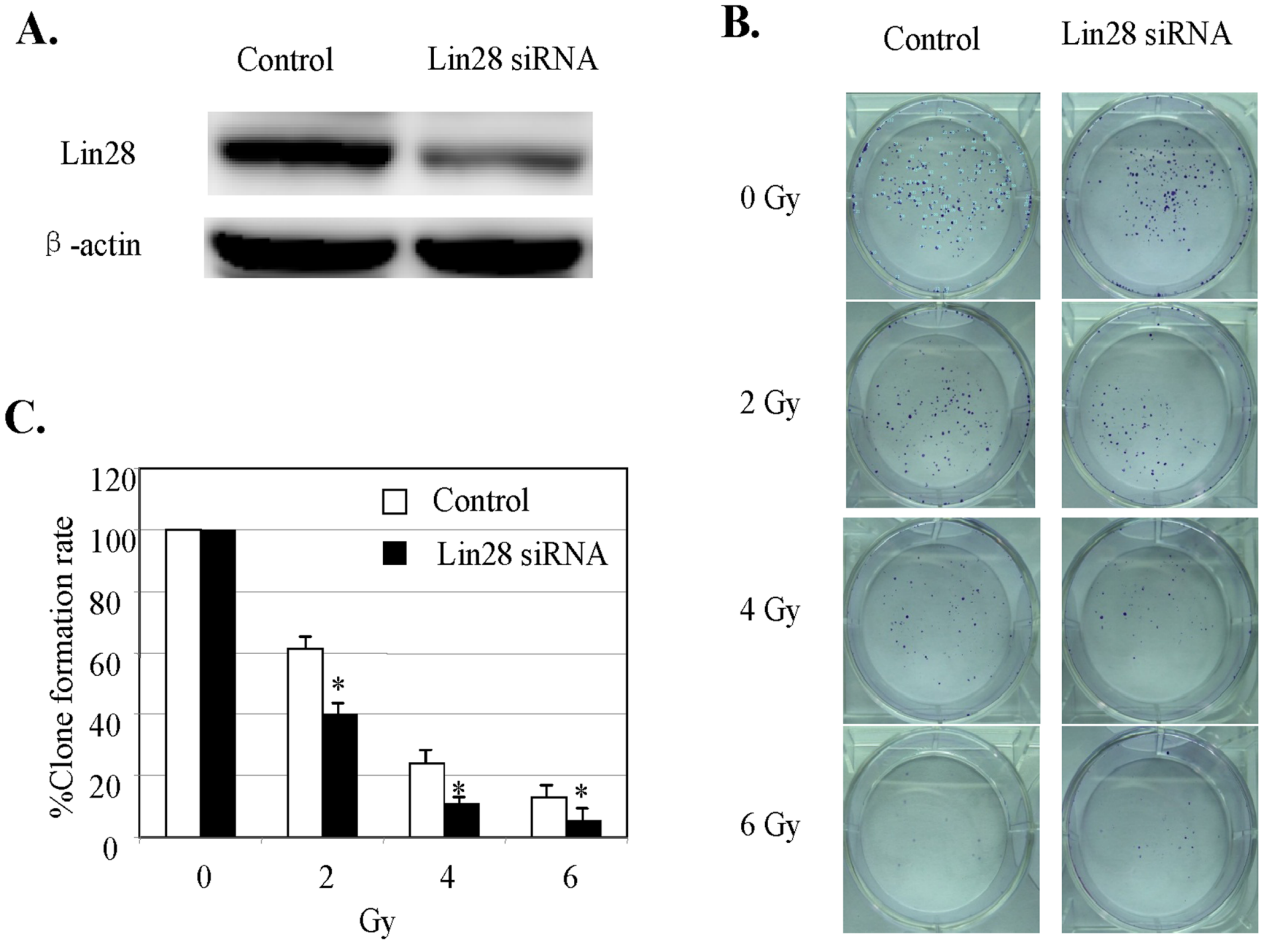
Lin28 knockdown by siRNA increases sensitivity to radiation. T47D cells with naturally high Lin28 expression were transfected with Lin28 siRNA and then were treated with radiation at the indicated doses. Cell viability was subsequently determined by the clonogenic formation assay (**B, C**). Lin28 expression in the indicated cell lines was determined by Western blotting (**A**). β-actin expression was used as a loading control. Each data point represents the mean ± SD of three independent experiments. *p<0.05.

To further confirm the role of Lin28 expression in regulating radiation resistance in breast cancer, we knocked down lin28 expression using Lin28 siRNA in T47D cancer cells with high Lin28 expression and determined their sensitivity to radiation. Compared with the control siRNA, transfection with Lin28 siRNA significantly decreased clonogenic formation in T47D cells treated with radiation at doses of 2 to 6 Gy, resulting in an increase in the sensitivity to radiation (Fig. 2B and 2C). The expression of Lin28 was confirmed by Western blotting (Fig. 2A).

**Figure 3:**
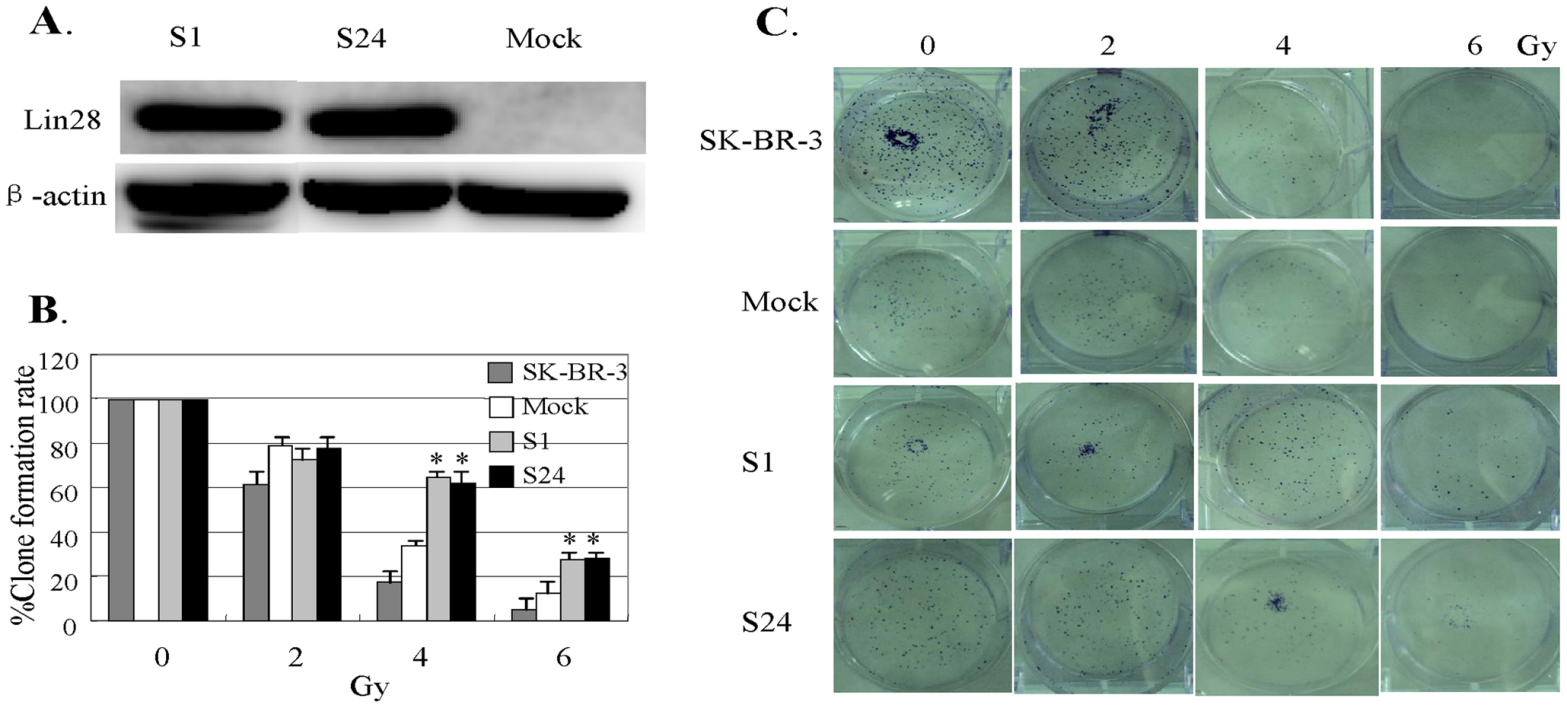
The SK-BR-3 cell line with stable Lin28 expression is relatively tolerant of radiation treatment. (**A**). Lin28 expression in the indicated cell lines was determined by Western blotting. β-actin was used as a loading control. (**B**). Cell viability of SK-BR-3 and its subclones S1 and S24, which stably express Lin28. Cells were treated with radiation, and cell viability was determined using the clonogenic formation assay. SK-BR-3 cells transfected with empty vector (SK-BR-3/mock) were used as a mock control. Each data point represents the mean ± SD of three independent experiments. *p<0.05. (**C**). Representative image of clone formation in the indicated cells after radiotherapy.

Next, we established SK-BR-3 clones (S1, S24) that stably expressed Lin28 and evaluated the sensitivities of these subclones to radiation. As shown in Fig. 3B and 3C, these two clones were more resistant to radiation compared with the control SK-BR-3 cells in the clonogenic formation assay. The expression of Lin28 was confirmed by Western blotting (Fig. 3A). These results confirm that the level of Lin28 expression was closely associated with the sensitivity of radiation.

### Lin28 Overexpression Inhibits Radiation-induced Apoptosis

**Figure 4:**
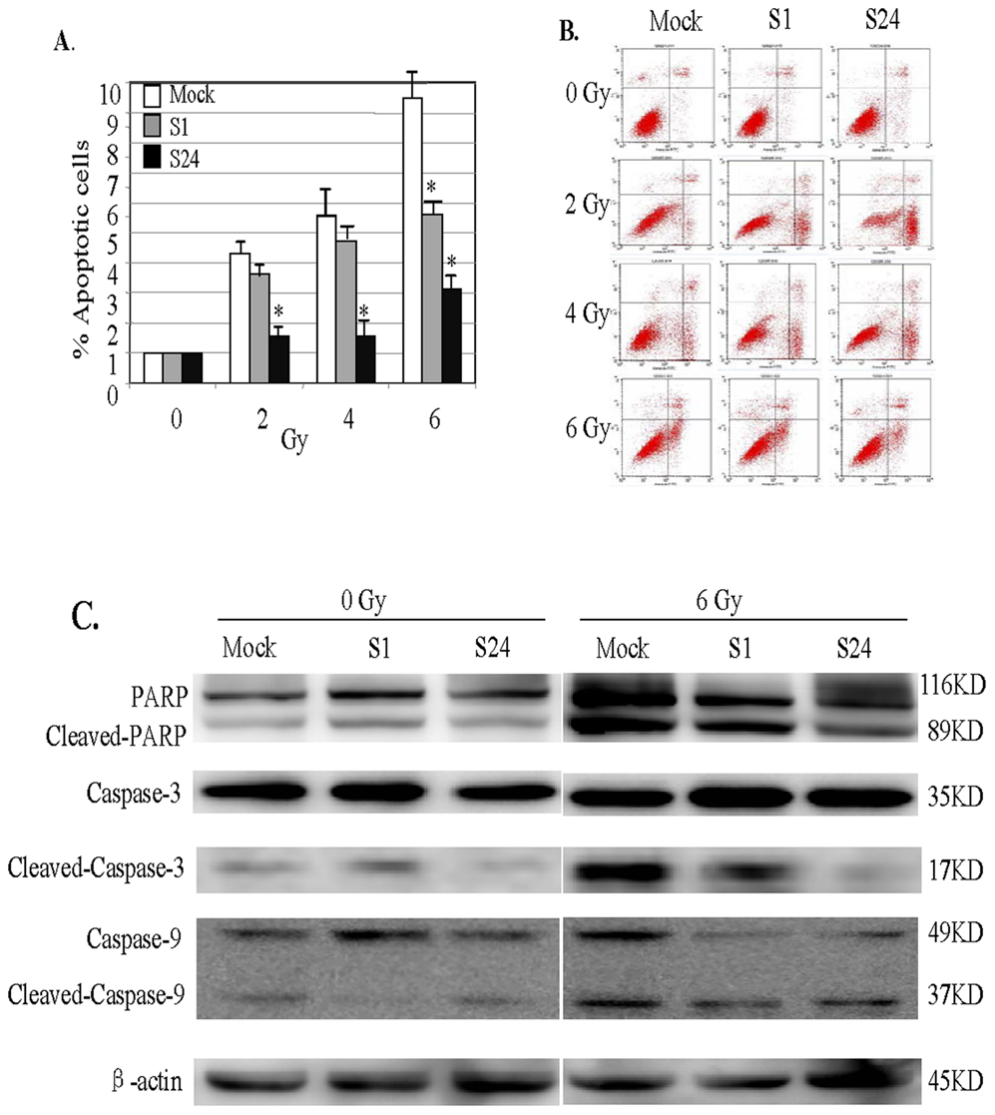
Overexpression of Lin28 decreases radiation-induced apoptosis in SK-BR-3 cell lines stably expressing Lin28. S1 and S24 cells were treated with radiation at doses ranging from 0 to 6 Gy, and apoptosis was analyzed. The empty vector-transfected cells were used as controls. (**A**)**.** Apoptosis compared with control. *, p<0.05. (**B**). Representative image of PI-Annexin V double staining examined in the S24 clone. (**C**). Expression of PARP, caspase-3 and caspase-9 proteins were determined in the indicated cells by Western blotting.

Radiotherapy has been shown to induce apoptosis in breast cancer cells. To determine whether Lin28-mediated redioresistance was induced by apoptotic inhibition, we next determined the effect of Lin28 overexpression on radiation-induced apoptosis. As shown in Fig. 4A and 4B, the two SK-BR-3 clones (S1 and S24) with stable Lin28 expression exhibited a significant reduction in radiation-induced apoptosis compared with control SK-BR-3 cells (P<0.05). To further confirm this, we also detected the activation of three key molecules involved in the apoptotic pathway: PARP, caspase-3 and caspase-9 proteins. The results showed that the cleavage levels of these three proteins were markedly up-regulated after radiation treatment in the mock control cells, whereas cleaved PARP, caspase-3, and caspase-9 levels were much lower in the S1 and S24 cells with stable Lin28 expression than the mock control cells (Fig. 4C). These results indicate that the inhibition of apoptosis contribute to the Lin28-mediated radiation resistance.

### Lin28 Induces H2A.X but Inhibits p21 andγ-H2A.X Expression

**Figure 5:**
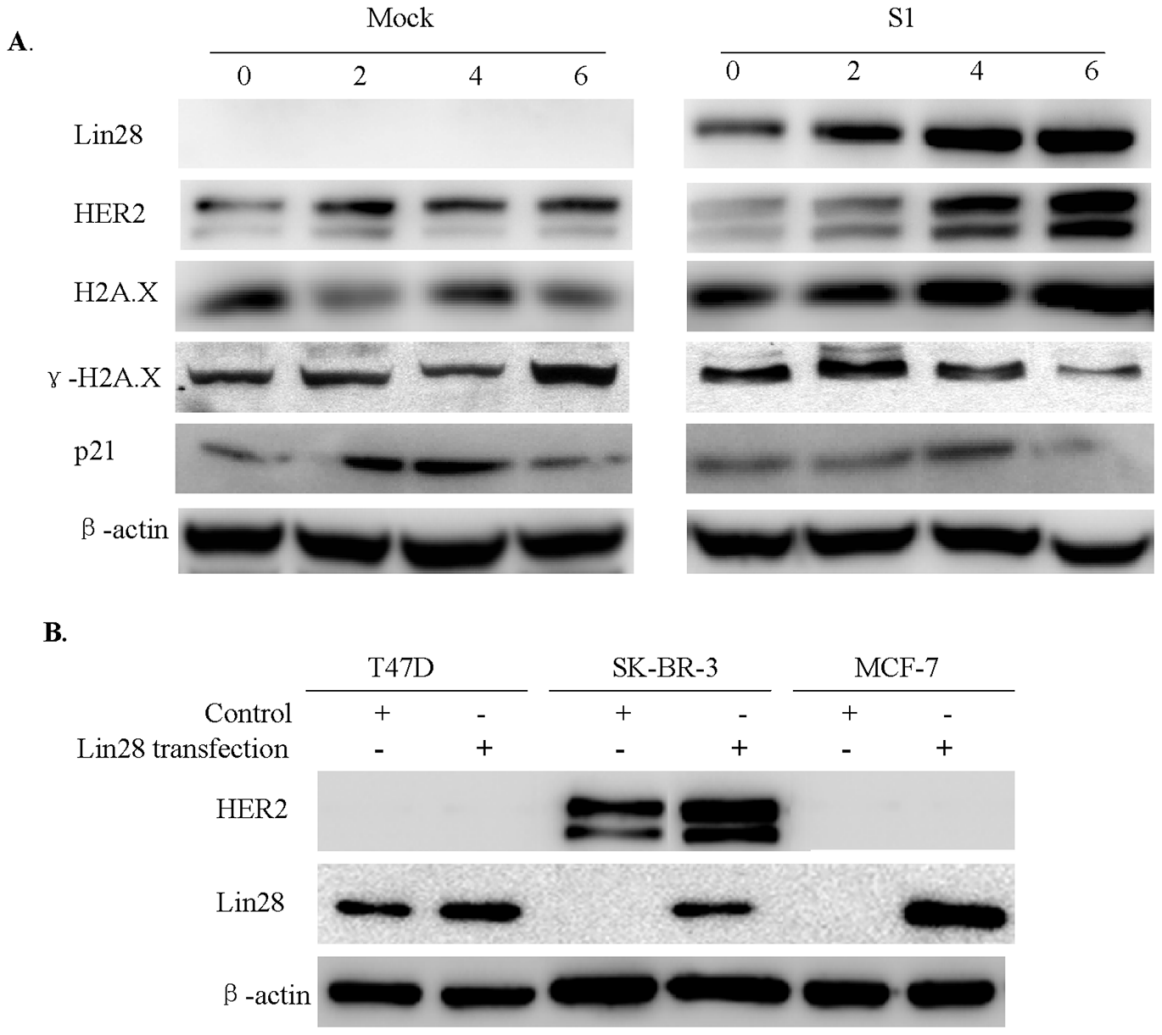
Lin28 inhibits expression of γ-H2AX and p21. (**A**). The expression of Lin28, HER2, H2A.X, γ-H2A.X and p21 in SK-BR-3 and S1 cells was determined 4 days after treatment of radiotherapy by Western blotting. β-actin was used as a loading control. (**B**). The expression of Lin28 and HER2 in indicated cells was determined by Western blotting.

To further investigate the mechanism of Lin28-induced radiation resistance, we measured the expression of three key genes–HER2, H2A.X, and p21–which are involved in breast tumorigenesis and radioresistance [9], in the three SK-BR-3 subclones that stably express Lin28. Following irradiation, Western blotting analysis showed that HER2 and H2A.X were significantly increased, while γ-H2A.X, as an active form of H2A.X, was significantly inhibited in cells stably expressing Lin28 compared with the mock controls, whereas p21 expression did not change (Fig. 5A).

To clarify whether HER2 is involved in Lin28-mediated radioresistance in breast cancer cells, we further test the HER2 expression in MCF-7, T47D and SK-BR-3 cell lines. The three cell lines were transfected with Lin28 vector, and test changement of HER2 expression. We found that HER2 expression was negative in MCF-7 and T47D cells, while HER2 higher level in SK-BR-3 cells. HER2 expression didn’t show significantly changement in Lin28 transfected groups compared to control groups transfected with empty vectors (Fig. 5B). These results indicated that HER2 was not involved in Lin28-mediated radioresistance in breast cancer cells.

### Let-7a Transfection Decreases Lin28-induced Radioresistance

**Figure 6:**
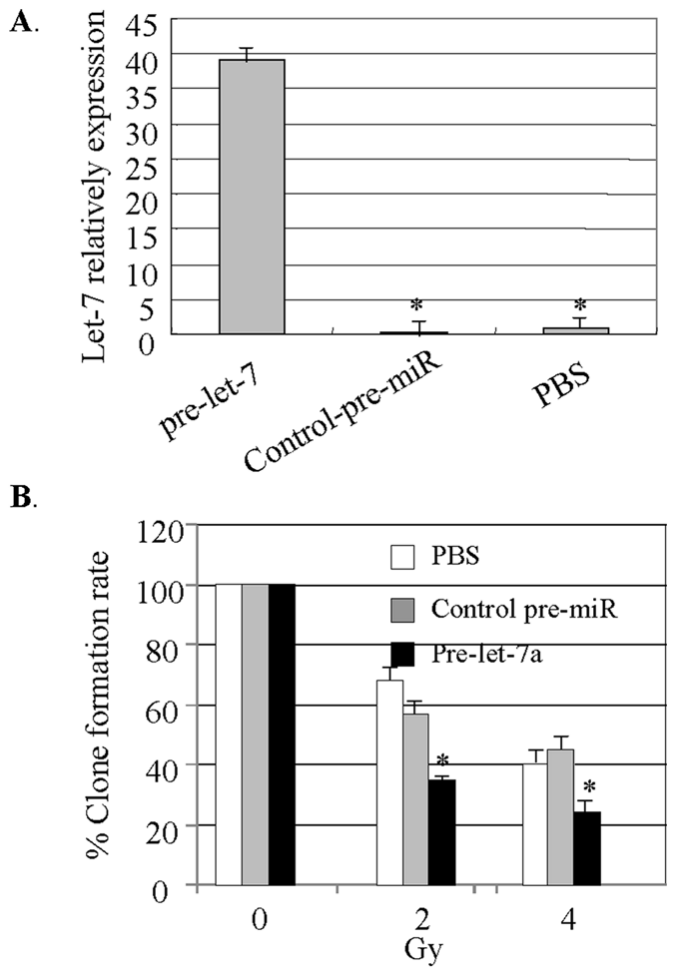
Let-7a overexpression decreases radioresistance in SK-BR-3 cell lines stably expressing Lin28. (**A**). Transfection of pre-let7a miRNA induced upregulation of Let-7a expression in S1 cells. Let-7a expression was determined by real-time qPCR. (**B**). S1 cells were treated with radiation, and cell viability was determined using the clonogenic formation assay. Cells transfected with control-pre-miR were used as a mock control. Each data point represents the mean±SD of three independent experiments. *p<0.05.

Let-7 miRNA has been shown to be regulated by Lin28 [10]. To further confirm the role of Lin28 expression in regulating radiation resistance in breast cancer, we next detected the association of Let-7 with radioresistance. As published previously, Let-7a and Let-7b miRNAs were dramatically decreased in the cancer cells stably expressing Lin28 compared with the mock controls [11]. The S1 cells were transfected with pre-Let-7a miRNA, and the effects of Let-7 on clonogenic formation were detected. As shown in Fig. 6B, overexpression of Let-7 dramatically enhanced the sensitivities of S1 cells to radiation compared with the control cells. The expression of Let-7a was confirmed by qPCR (Fig. 6A). These results show that let-7 miRNA downregulation could be one of the mechanisms of Lin28-induced radioresistance in breast cancer cells.

## Discussion

In the present study, we investigated the association of Lin28 expression with radioresistance in breast cancer cells. We found that Lin28 expression was up-regulated in radiation-resistant breast cancer cells and that Lin28 transfection induced radiation-resistance in breast cancer cells. We also showed that overexpression of Lin28 and radiation effectively induced H2A.X expression, inhibited H2A.X active formγ-H2A.X and p21 expression, and overexpression of Let-7 enhanced the sensitivity to radiation. To our knowledge, this is the first finding that demonstrates that Lin28 expression is a possible mechanism of radioresistance in breast cancer and suggests that Lin28 could be a potential target to overcome radioresistance in breast cancer.

Radiotherapy is one of the major treatments for patients with cancers. However, radioresistance remains a major factor that limits the effectiveness of radiotherapy. Therefore, identification of the tumor response to radiotherapy has long been a goal of oncologists and biologists. In the last century, scientists have observed an association between radioresistance and the expression of several candidate genes, such as EGFR [12], AKT [13], P53 [14], HER2 [9], HIF-1 [15] and Bcl-2 [16]. Recently, cancer stem cells have emerged as a contributor to radioresistance through the preferential activation of the DNA damage checkpoint response and an increase in DNA repair capacity [17]–[19]. Furthermore, changes in some miRNA levels have been shown to be associated with radiotherapy sensitivity [8]; [20]; [21]. Previous studies have shown that Lin28 is overexpressed in hepatocellular carcinoma, overexpression of the Lin28 gene promotes cancer cell proliferation in vitro [5], and a relationship exists between the expression of Lin28 and the radioresistance of lung pancreatic cancer cells [8]. However, no report has shown a relationship between the dysregulation of Lin28 and the radioresistance of breast cancer cells. In the present study, we explored the possible mechanisms of action of Lin28.

In summary, we showed that Lin28 mediates radiation resistance by modulating H2A.X, and caspase-dependent signaling pathways in breast cancer cells. We also demonstrated that Let-7 overexpression reduced the radiation resistance. Our study therefore indicates Lin28 may be a potential target to overcome radioresistance in breast cancer treatment.

## Materials and Methods

### Cell Culture and Chemicals

Human breast cancer cell lines (SKBR-3, MCF7, Bcap-7, MDA-231, T47D) were obtained from the American Type Culture Collection (ATCC, Manassas, VA) and cultured in Dulbecco’s modified Eagle’s Medium (DMEM) supplemented with 10% heat-inactivated fetal calf serum, 100 units/mL penicillin, and 100 mg/mL streptomycin (Invitrogen, Carlsbad, CA). In all the experiments, cells were grown at 37°C in an atmosphere of 5% CO_2_.

### Clonogenic Assays

Cells in the 10 cm dishes irradiated with various doses of radiation in a ^137^Cs unit at room temperature. The irradiated cells were then seeded in triplicate 10 cm dishes at a density of 1000 cells per dish to yield 50 colonies per dish. The cells were then cultured in an incubator containing 5% CO_2_ at 37°C for 14 to 21 days. Individual colonies (>50 per colony) were fixed and stained with a solution containing crystal violet and 10% ethanol for 10 minutes. The colonies counted with an imaging system (FluorChem 8800 Imaging System Alpha Innotech, San Leandro, CA) using a visible light source. Each experiment was done in triplicate and repeated at least twice.

### Transfection

Cells were plated in six-well culture plates and transfected with 50 nM of siRNA purchased from Applied Biosystems (Carlsbad, CA) or control siRNA according to the manufacturer’s protocol.

### Quantitative Real-time Polymerase Chain Reaction (PCR)

Real-time PCR was performed using the TaqMan MicroRNA Reverse Transcription Kit and the Fast Real-Time PCR System (Applied Biosystems) according to the manufacturer’s protocols. The fold change of Let-7a microRNA levels was calculated and normalized to an hsa-mir-423 loading control.

### Western Blot Analysis

Cell lysates were separated by electrophoresis on a 4–15% sodium dodecyl sulfate-polyacrylamide gradient minigel (SDS-PAGE) (Bio-Rad, Hercules, CA) and electrophoretically transferred to a nitrocellulose membrane (Amersham Pharmacia, Piscataway, NJ). Western blots were probed with antibodies against Lin28,HER2, p21 (Santa Cruz Biotechnology, Santa Cruz, CA), AKT (Cell Signaling, Beverly, MA) or β-actin (Sigma, St. Louis, MO). The protein bands were detected by enhanced chemiluminescence (Amersham Pharmacia, Piscataway, NJ).

### Flow Cytometric Assay

Cells (2×10^5^ per well) were plated in a 6-cm plate and treated with radiation. After 72 h, cells were stained with FITC Annexin V and propidium iodide. The cells were analyzed using an Epics Profile II flow cytometer (Beckman Coulter, Fullerton, CA) and Multicycle software (Phoenix Flow Systems, San Diego, CA). All the experiments were repeated at least twice.

### Statistical Analysis

All the experiments were performed three times with triplicate samples. Analysis of variance and Student’s *t*-test were used to compare the values of the test and control samples. A P-value less than 0.05 was deemed to be statistically significant. Statistica 6.1 software was used for all statistical analyses. The significance was evaluated by the paired *t*-test.
